# Seronegative Immunoglobulin G4‐related hypertrophic pachymeningitis misdiagnosed as meningitis and meningioma

**DOI:** 10.1002/kjm2.12816

**Published:** 2024-02-16

**Authors:** Meng‐Leo Chou, Hui‐Chun Chen, Shih‐Huang Tai, Chin‐Wei Huang

**Affiliations:** ^1^ Department of Neurology, National Cheng Kung University Hospital, College of Medicine National Cheng Kung University Tainan Taiwan; ^2^ Department of Pathology, National Cheng Kung University Hospital, College of Medicine National Cheng Kung University Tainan Taiwan; ^3^ Division of Neurosurgery, Department of Surgery, National Cheng Kung University Hospital, College of Medicine National Cheng Kung University Tainan Taiwan

Immunoglobulin G (IgG)‐4‐related hypertrophic pachymeningitis represents a rare manifestation of IgG4‐related disease (IgG4‐RD), characterized by a multi‐organ fibroinflammatory disorder featuring tumefactive lesions with distinctive pathological features. Typically, this condition manifests through localized or diffuse thickening of the dura mater, and in some instances, tumor‐like lesions have been documented in various cases^1^, ^2^ Such presentations can contribute to misdiagnosis, potentially resulting in incomplete investigation and suboptimal follow‐up plans.

A 65‐year‐old Chinese woman presented at our neurology outpatient department with bouts of focal seizures. Her medical history revealed dyslipidemia and coronary artery disease. Seven years ago, she experienced left hemibody tingling numbness for months, attributed to a “mass lesion over the right parietal lobe.” Subsequently, she underwent surgical resection at a medical center, and the pathological report described it as “meningitis” without a specific diagnosis. Sensory deficits improved, and there were no further treatment or regular follow‐up plans.

Six years later, she began experiencing episodes of focal seizures characterized by leftward head turning, left limb tonic–clonic movements, and trismus. Neurological examination revealed left hemibody hypoesthesia. Electroencephalography revealed an alpha rhythm background with occasional medium amplitude of focal theta activities at bilateral anterior and medial temporal areas, including intermittent quasi‐rhythmic sharply contoured theta activities over the left temporal area. Brain magnetic resonance imaging (MRI) revealed a 3.9‐cm Gadolinium‐enhancing dura‐based tumor at the right frontal convexity with a typical “dural tail sign” (Figure 1A,B), leading to a clinical impression of meningioma.

**FIGURE 1:**
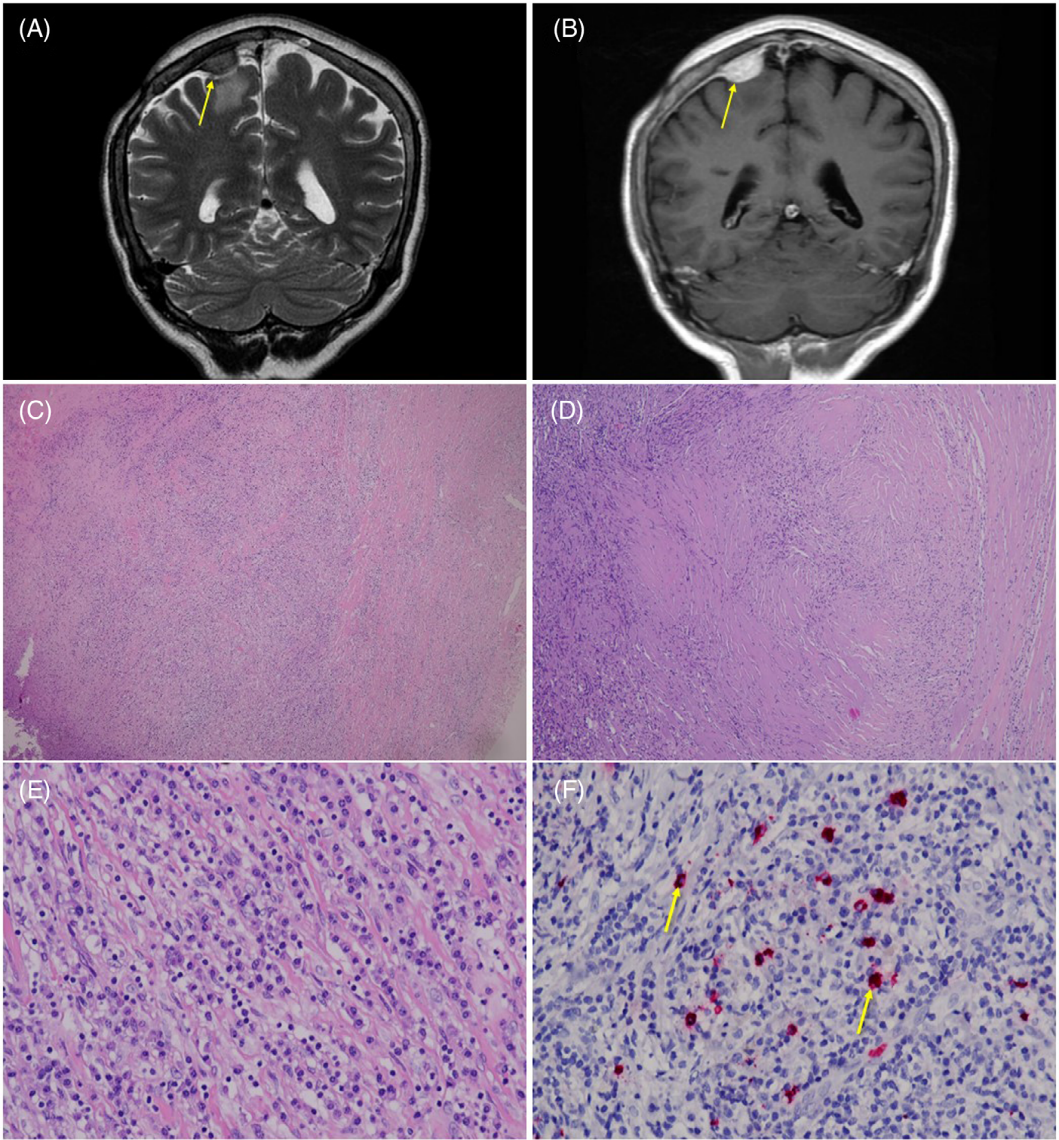
Initial brain magnetic resonance imaging (MRI) shows (A) a dura‐based mass lesion at right frontal convexity with perifocal edema on T2‐weighted image and (B) intense, heterogeneous Gadolinium‐enhancement on T1 image (arrows). Histopathological assessment reveals (C) fibrosis and dense inflammatory cells infiltrate and (D) irregularly storiform fibrosis under low power view. (E) Higher power view demonstrates that the inflammatory cells are predominantly composed of lymphocyte and plasma cells. (F) The IgG4 immunostain shows increase IgG4‐positive plasma cells (around 20 cells per high‐power filed; arrows).

Initially, phenytoin was administered for seizures, later changed to zonisamide due to dermal allergic reactions. To investigate and treat the tumor comprehensively, surgical resection was performed. Histopathological evaluation revealed thick collagen bundles with dense lymphoplasmacytic infiltration, including neutrophils and eosinophils. Suppurative granulomatous inflammation was evident, while no vasculitis was observed.

Immunohistochemically, actin demonstrated some myofibroblasts, and epithelial membrane antigen‐stained plasma cells. IgG4‐positive cells accounted for ~20 cells per high‐power field (Figure 1C–F).

The diagnosis of meningioma was reconsidered in light of the results from the histopathological assessment and the patient's complete clinical history, which included her previous surgery. The patient's serum IgG4 level was within the normal range (22.8 mg/dL). Subsequently, a diagnosis of probable IgG4‐related hypertrophic pachymeningitis was established, based on the revised comprehensive diagnostic criteria for IgG4‐related disease.^3^ A systematic approach to managing this disease has been initiated.^4^, ^5^

The patient was later discovered to have an abdominal aortic aneurysm, in addition to her existing coronary artery disease. While immunotherapy was suggested, it had not been administered due to the patient's concerns. The focal‐onset seizures showed improvement, occurring at a low frequency of once every several months under regular use of zonisamide. Annual repeat brain MRI scans have not revealed a recurrence of the space‐occupying lesion. Regular systemic follow‐up has been diligently conducted.

The case report presents a meningioma‐mimicking pseudotumor initially overlooked in the disease course, diagnosed as probable IgG4‐related hypertrophic pachymeningitis. This case features a distinctive clinical presentation accompanied by characteristic histopathological findings. We share this case to heighten awareness that IgG4‐related hypertrophic pachymeningitis should be considered as a significant differential diagnosis for meningioma, particularly in cases involving seizures, showcasing the diverse clinical spectrum of IgG4‐RD. A prompt and accurate diagnosis of IgG4‐RD is crucial for facilitating comprehensive systemic investigations and expediting appropriate treatment.

## CONFLICT OF INTEREST STATEMENT

The authors declare no conflicts of interest.
